# AQP1-Driven Migration Is Independent of Other Known Adverse Factors but Requires a Hypoxic Undifferentiated Cell Profile in Neuroblastoma

**DOI:** 10.3390/children8010048

**Published:** 2021-01-15

**Authors:** Nicola Pini, Zihe Huo, Urs Kym, Stefan Holland-Cunz, Stephanie J. Gros

**Affiliations:** 1 Department of Pediatric Surgery, University Children’s Hospital Basel, 4031 Basel, Switzerland; nicolanpini@gmail.com (N.P.); zihe.huo@ukbb.ch (Z.H.); urskym@hotmail.com (U.K.); stefan.holland-cunz@ukbb.ch (S.H.-C.); 2 Department of Clinical Research, University of Basel, 4031 Basel, Switzerland

**Keywords:** aquaporin 1, tumor heterogeneity, neuroblastoma, protein function, protein interaction, HIF, NMYC, NCAM, CXCR4

## Abstract

Neuroblastoma is a biologically very heterogeneous tumor with its clinical manifestation ranging from spontaneous regression to highly aggressive metastatic disease. Several adverse factors have been linked to oncogenesis, tumor progression and metastases of neuroblastoma including NMYC amplification, the neural adhesion molecule NCAM, as well as CXCR4 as a promoter of metastases. In this study, we investigate to what extent the expression of AQP1 in neuroblastoma correlates with changing cellular factors such as the hypoxic status, differentiation, expression of known adverse factors such as NMYC and NCAM, and CXCR4-related metastatic spread. Our results show that while AQP1 expression leads to an increased migratory behavior of neuroblastoma cells under hypoxic conditions, we find that hypoxia is associated with a reduction of NMYC in the same cells. A similar effect can be observed when using the tetracycline driven mechanism of SH-EP/Tet cells. When NMYC is not expressed, the expression of AQP1 is increased together with an increased expression of HIF-1α and HIF-2α. We furthermore show that when growing cells in different cell densities, they express AQP1, HIF-1α, HIF-2α, NMYC and NCAM to different degrees. AQP1 expression correlates with a hypoxic profile of these cells with increased HIF-1α and HIF-2α expression, as well as with NMYC and NCAM expression in two out of three neuroblastoma cell lines. When investigating cell properties of the cells that actually migrate, we find that the increased APQ1 expression in the migrated cells correlates with an increased NMYC and NCAM expression again in two out of three cell lines. Expression of the tumor cell homing marker CXCR4 varies between different tumor areas and between cell lines. While some migrated tumor cells highly express CXCR4, cells of other origin do not. In the initial phase of migration, we determined a dominant role of AQP1 expression of migrating cells in the scratch assay.

## 1. Introduction

Neuroblastoma is a biologically very heterogeneous tumor with clinical manifestations ranging from spontaneous regression to highly aggressive metastatic disease that is unresponsive to standard and investigational anti-cancer treatment [1]. The tumors develop mostly in the adrenal medulla or other locations with an origin of the neural crest, like the paraspinal sympathetic ganglia or the pelvic ganglia. This is reflected in the fact that many genes that are involved in the regulation of neural crest development are also expressed in neuroblastoma [2]. Neuroblastoma cells can differentiate and assume phenotypes that range from an initial epithelial-like to a more branched and neuronal one [3]. There are many different surface markers whose expression varies depending on the differentiation stage of the cells. To classify the tumor into four risk categories, the International Neuroblastoma Risk Group (INRG) classification system is based on factors as age at diagnosis, tumor stage, histology, differentiation grade, copy number status of NMYC and deletion status of chromosome 11q [4].

Major adverse factors include amplification of NMYC, expression of the neural cell adhesion molecule NCAM and of metastatic homing chemokine receptor CXCR4. NMYC is known to be essential for the differentiation state of neuroblastoma cells and the fate of neural crest cells [5,6]. Its knockdown leads to apoptosis and terminal differentiation of the cells [7]. However, NMYC is expressed neither in all neuroblastoma tumors nor in all neuroblastoma cell lines. In recent years many different surface markers of tumor cells have been identified and some of them are used to determine the malignancy and the metastatic potential of neuroblastoma [8]. The neural cell-adhesive molecule (NCAM/CD56) is such a surface molecule and belongs to the immunoglobulin superfamily. NCAM is expressed in many tumor types and is associated with increased malignancy, increased metastatic potential and an overall more aggressive behavior of the tumors leading to a worse prognosis [9,10,11]. Chemokine receptors represent a large family of cytokine receptors. Among these, CXCR4 is one of the most studied because of its expression by many types of cancer cells [12]. It has been shown that a high expression of CXCR4 in neuroblastoma correlates with a significantly impaired outcome compared to a low CXCR4 expression [13,14].

Aquaporins (AQP) are a family of transmembrane proteins expressed in many endothelial and epithelial tissues, as well as in the peripheral and central nervous system [15]. They have the capacity to transport water and play a role in cell migration and adhesion [16]. Especially AQP1, the first water channel described [17], is expressed in several tumor tissue and has been linked with the promotion of migration [16,18]. We have previously shown that AQP1 expression leads to an increased migratory behavior of neuroblastoma cells through its up-regulation under hypoxic conditions (Huo, to be published). We hypothesized that during a hypoxic window cells are transforming towards an undifferentiated, migratory phenotype with an increased AQP1 expression. The influence of HIF-1α and HIF-2α on the differentiation of neuroblasts is controversially discussed [19,20,21]. However, it was shown that hypoxia led to a de-differentiation that persisted for 24 h after re-oxygenation [22]. Jogi et al. showed that hypoxic neuroblastoma cells are transformed into a more immature and neural crest-like phenotype and thus contribute to malignancy [23,24]. Hypoxia dependent differentiation status of the cells could be a crucial factor in the migration process. One challenge of investigating neuroblastoma is that the numerous existing neuroblastoma cell lines, as well as patient tumors, show great heterogeneity regarding not only AQP1 expression but also the expression of other known factors that are associated with a worse outcome or a higher malignant potential. In this study, we investigate to what extent the expression of AQP1 in neuroblastoma correlates with or is dependent on changing cellular factors such as differentiation, expression of know adverse factors such as NMYC and NCAM, and metastatic spread related to CXCR4.

## 2. Materials and Methods

### 2.1. Cell Culture

Neuroblastoma Kelly, SH-SY5Y (all ECACC/Sigma-Aldrich, Munich, Germany) and SH-EP Tet-21/N cells (reported by Lutz et al. [25,26], kindly provided by G. Eschenburg, Hamburg) were cultivated in RPMI media containing 10% FCS. If possible, aliquots of early passages (4–6) after purchase were used for all experiments. All cells were cultured in a humidified atmosphere at 37  °C either in air with 5% CO_2_ under normoxic or with 1% O_2_ balanced with N_2_ with 5% CO_2_.under hypoxic conditions. For using the MYCN off mechanism 1μg/mL tetracycline was added to the medium for 72h. For cell differentiation, the differentiation protocol by Shipley et al. was applied [3]. For investigation regarding the cell density, cells were seeded in chamber slides (Thermofisher, Corning, NY, USA) at low (Kelly and SH-SY5Y-1 × 10^5^ cells/mL; SH-EP Tet21/N cells-1 × 10^4^ cells/mL), middle (Kelly and SH-SY5Y-5 × 10^5^ cells/mL; SH-EP Tet21/N cells-5 × 10^4^ cells/mL), highly (Kelly and SH-SY5Y-1 × 10^6^ cells/mL; SH-EP Tet21/N-1 × 10^5^ cells/mL) dense cell concentrations and incubated for 24 h at normoxic conditions.

### 2.2. Tumor Tissue

Patient tumor samples resected in the course of the regular treatment, and only resected material that was not needed for clinical investigations, was used after patient consent, as approved by the appropriate ethics committee (Ethikkommission Nordwest- und Zentralschweiz EKNZ 2015-263). Tumor tissue was taken from the resected specimen immediately after surgery and snap-frozen. Tissues were cut using a cryotome and dissected into tissue pieces for RNA analysis.

### 2.3. In Vitro Wound Healing

Cells were cultured as confluent monolayers, synchronized in 1% fetal bovine serum for 24 h, and wounded by removing a defined 300–500 µm strip of cells across the well with a standard 200 µL pipette tip [27]. The wounded monolayers were washed twice to remove non-adherent cells. Wound healing was quantified as the average linear speed of the wound edges over 24 h. To eliminate possible artifacts produced by differences in proliferation, wild type and AQP1-modified cells were compared regarding proliferation and no differences were found (data not shown).

### 2.4. Modified Migration Assay

Tumor cell migration through a microporous membrane was assessed using a Boyden transwell system (8 µm pore size, Corning Costar, NY, USA). Before seeding, transwell systems were incubated in RPMI-media containing 2% FCS for 30 min at room temperature. An amount of 200 µL RPMI-media with 2% FCS containing dissociated cells (3 × 10^4^ per well) was added to the upper insert of the chamber. In the bottom chamber, 500 µL RPMI-media with 10% FCS was added. Twenty-four hours after seeding, cells of the upper and lower chambers were harvested and placed on ice. Messenger RNA was retrieved and further processed as described below. Experiments were performed in triplicates and repeated.

### 2.5. RNA Isolation/cDNA Synthesis/qPCR

Cells were harvested, washed with ice-cold PBS and then lysed in the buffer RLT Plus (QIAGEN). RNA isolation was subsequently performed using the RNeasy Plus Mini (QIAGEN, Cat.No. 74134) or Micro (QIAGEN, Cat.No. 74034) Kit, for more or less than 5 × 10^5^ cells, respectively. RNA isolation was performed according to the manual, and RNA was eluted in 30 µL or 14 µL nuclease-free water, respectively. RNA concentration was determined using a Colibri Microvolume Spectrometer (BioConcept AG, Allschwil, Switzerland). CDNA synthesis was performed using the GoScript™ Reverse Transcription System (Promega, Cat.No. A5000). A Biometra T-Personal Thermal Cycler (Analytik Jena, Jena, Germany) was used.

Quantitative PCR was performed using the FastStart Universal SYBR Green Master (Rox) (Roche, Cat.No. 4913850001). AQP1, HIF-1α, HIF-2α, CXCR4, NMYC, NCAM, Ki67 and 18S specific primers (Microsynth, Balgach, Switzerland) were used for amplification of cDNA. Primer pairs were designed according to exon junction span using the clone manager software (Sci-Ed Software Colorado, USA). Per reaction, 5 µL Master Mix were mixed with primers fwd and rev (0.5 µM), 1 µL of cDNA template or water in NTC, and water to a final volume of 10 µL. Furthermore, 18s was used as a reference gene. Reactions were performed in triplicates in MicroAmp™ Optical 384-Well Reaction Plates (Applied Biosystems, Cat.No. 4309849) in a ViiA 7 Real-Time PCR System (Applied Biosystems) using the associated software. Data were analyzed using the 2^−ΔCT^ method × 1000 and depicted in Excel (Microsoft Office 2010) and GraphPad Prism 7. Relative expression was calculated against expression of 18 s.

### 2.6. Immunofluorescence Staining

For AQP1, MAP2 and CXCR4 immunofluorescence staining, the polyclonal rabbit anti-AQP1 antibody (Merck Millipore, Darmstadt, Germany), the monoclonal mouse MAP2 antibody (Abcam, Cambridge, UK) and the polyclonoal goat CXCR4 antibody (Abcam, Cambridge, UK) were used at a dilution of 1:400, 1:200, and 1:100 respectively following a standardized protocol. As secondary antibodies goat anti-mouse IgG1 AlexaFluor 488 and goat anti-rabbit AlexaFluor 647 were used. Negative controls missing the primary antibody were included in every staining cycle. Slides were mounted using ProLongVR Gold Antifade Mountant with DAPI (Life Technologies, Thermo Fisher Scientific, Waltham, MA, USA). Slides were analyzed with an Olympus BX43 microscope using CellSens software.

### 2.7. Flow Cytometry

For FACS staining, cells were centrifuged in a 96 well plate (all centrifugation steps were performed at 280× *g*, 3 min. at RT) and re-suspended in 50 µL PBS containing 1/1000 LIVE/DEAD^®^ Fixable Near-IR Dead Cell Stain (ThermoFisher Scientific, Cat.No. L-10119). Cells were stained for 5 min. on ice and washed with PBS. Afterward, cells were re-suspended in 50 µL FACS-Buffer (PBS + 2–3% heat-inactivated FCS (BioConcept AG, Allschwil, Switzerland, Cat.No. 2-01F10-I)) + 1/100 Fc-blocking antibody containing the primary antibodies polyclonal rabbit anti-AQP1 antibody (Merck Millipore, Germany) and anti-CD56-PerCP (NCAM, Biolegend, CA, USA). Cells were stained for 15 min on ice and washed with FACS Buffer. Secondary antibody anti-rabbit Alexa 647 was added in 50 µL FACS-Buffer at appropriate concentrations, cells were stained for 25 min. on ice and washed with FACS-Buffer. Cells were re-suspended in an appropriate amount of FACS-Buffer and transferred into 5 mL tubes. Negative controls were included for each antibody. Samples were measured using FACSCanto II cytometry system (BD Bioscience).

### 2.8. Statistical Analysis

Data were analyzed using the SPSS version 23.0 (SPSS, Chicago, IL, USA), Excel and GraphPad Prism 7 Software. For significance testing, the two-sided *t*-test or analysis of variance with a post-hoc test was used. *p* values less than 0.05 were defined as significant. The error bars in all bar plots represent one standard deviation.

## 3. Results

### 3.1. Tumor Heterogeneity

**Figure 1:**
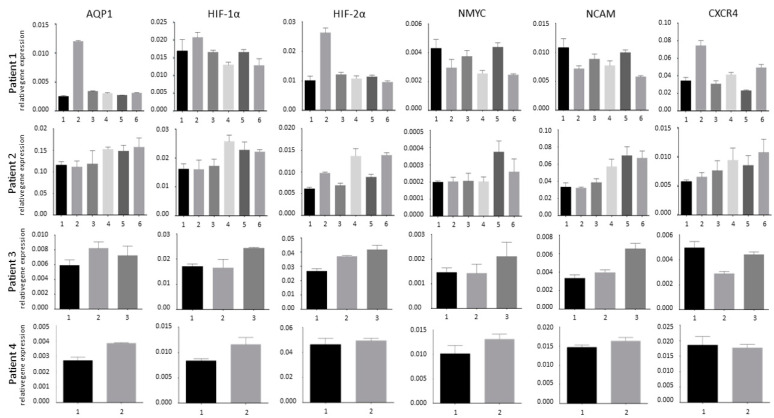
Inter-and intra-patient heterogeneity.

We investigated the gene expression profile of different tumor areas of four patients that were treated in our clinic and underwent either total/subtotal resection or biopsy for neuroblastoma. Depending on tumor size we analyzed two to six tissue areas regarding the RNA expression properties with respect to their hypoxia-dependent factors as well as NMYC, NCAM and CXCR4 (Figure 1). Our data demonstrate that even within one tumor there is impressive heterogeneity. Patient 1 presented with the primary tumor in a paravertebral location. The stage 1 tumor was resected primarily as a resection biopsy. Tumor tissue of patient 2 originates from a progressive tumor at the primary site after preceding subtotal resection of the original stage 3 tumor. In patient 3, the residue of the primary tumor at an adrenal location was resected in the course of the treatment in congruence with the SIOPEN protocol. Tumor tissue of patient 4 was harvested in the course of a biopsy at an abdominal location. In routine pathological evaluation, FISH analysis was negative for NMYC amplification in all four tumors. Although patient 3 was the only patient that had successfully been treated with chemotherapy prior to resection, the differences in marker expression between different tumor areas were still pronounced. The tissue of patient 4 originated from a small biopsy, therefore the area of the tumor that we were able to examine was restricted. This might also be the reason for the fact that the two examined areas are rather similar. Gene expression analysis of AQP1, HIF-1α, HIF-2α, CXCR4, NMYC, NCAM mRNA reveals major differences between tumors but also between different areas of the same tumor, demonstrating great inter- and intra-tumor heterogeneity. A correlation of AQP1 with the hypoxic key regulators HIF-1α and/or HIF-2α can be observed in several pieces in all patients.

Relative expression of AQP1, HIF-1α, HIF-2α, CXCR4, NMYC, NCAM mRNA in relation to the housekeeping gene 18s (*y*-axis, relative expression in relation to 18s) in diverse pieces (*x*-axis, pieces 1–6) of four different patient tumors is shown. Differences in clinical presentation and heterogeneous gene expression patterns of different areas demonstrate the great inter- and intra-tumor heterogeneity. A correlation of AQP1 with the hypoxic key regulators HIF-1α and/or HIF-2α can be observed in several pieces in all patients.

In the cell culture setting, we often observe a similar heterogeneity within one cell culture dish. Cells in the same culture can present at different differentiation stages. Moreover, different cell lines are characterized by different properties. In this study, we investigate the relationship of AQP1 expression with other adverse factors in neuroblastoma. We previously hypothesized that AQP1 further migrations and aggressive tumor cell behavior (Huo, to be published). In the light of the know inter- and intra-tumor heterogeneity we chose three cell lines with different properties for our experiments, in order to study this heterogeneity. Criteria for cell line selection were their AQP1 expression status, the expression of the established and clinically used markers for tumor aggressiveness and outcome NMYC and the expression neuronal crest adhesion marker (NCAM).

SH-EP Tet-21/N cells were derived from the non-NMYC amplified SH-EP cell line and present a metastatic subclone [25,26]. They are characterized by a tetracycline driven NMYC on/off mechanism. In the presence of tetracycline they cease to express NMYC. Furthermore, SH-EP Tet-21/N cells do not express AQP1 under normoxia or hypoxia to any relevant degree (Huo, to be published) and are NCAM negative (data not shown). Kelly and SH-SY5Y cells are the most commonly used cell lines. They both express AQP1 under hypoxia (Kelly > SH-SY5Y). Kelly cells are NMYC positive while SH-SY5Y cells are NMYC negative. NCAM is expressed in both cell lines to varying degrees. SH-SY5Y cells can be differentiated in cell culture using an established protocol [3].

### 3.2. Hypoxia and Unfavorable Properties

**Figure 2:**
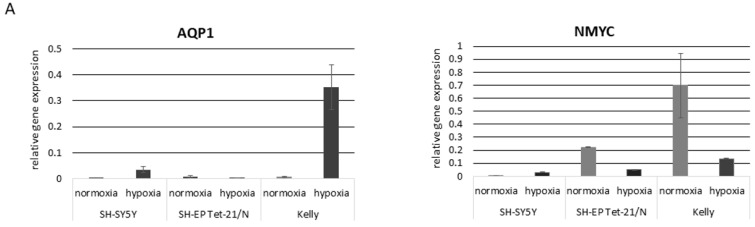

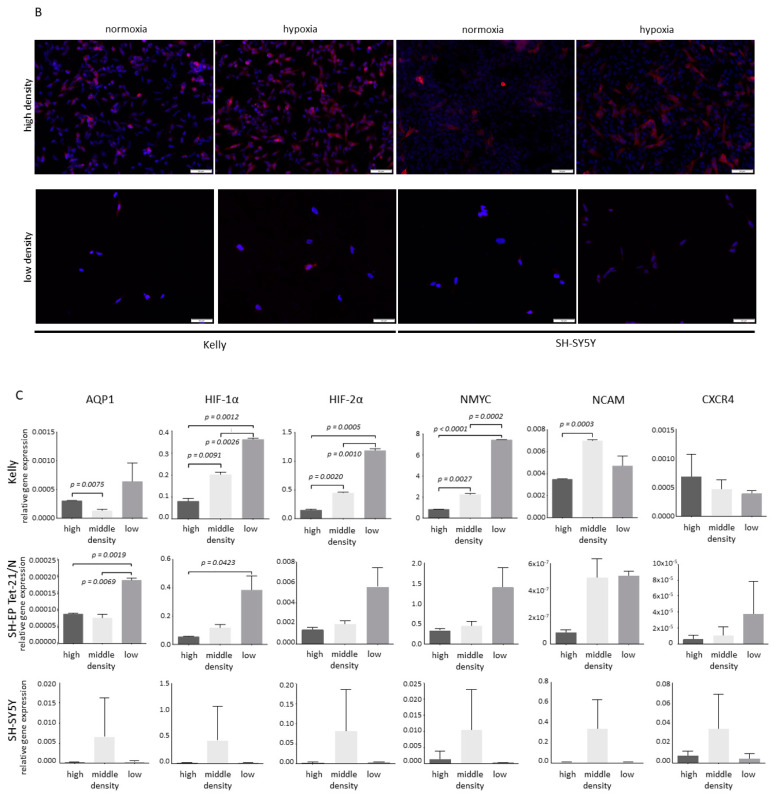

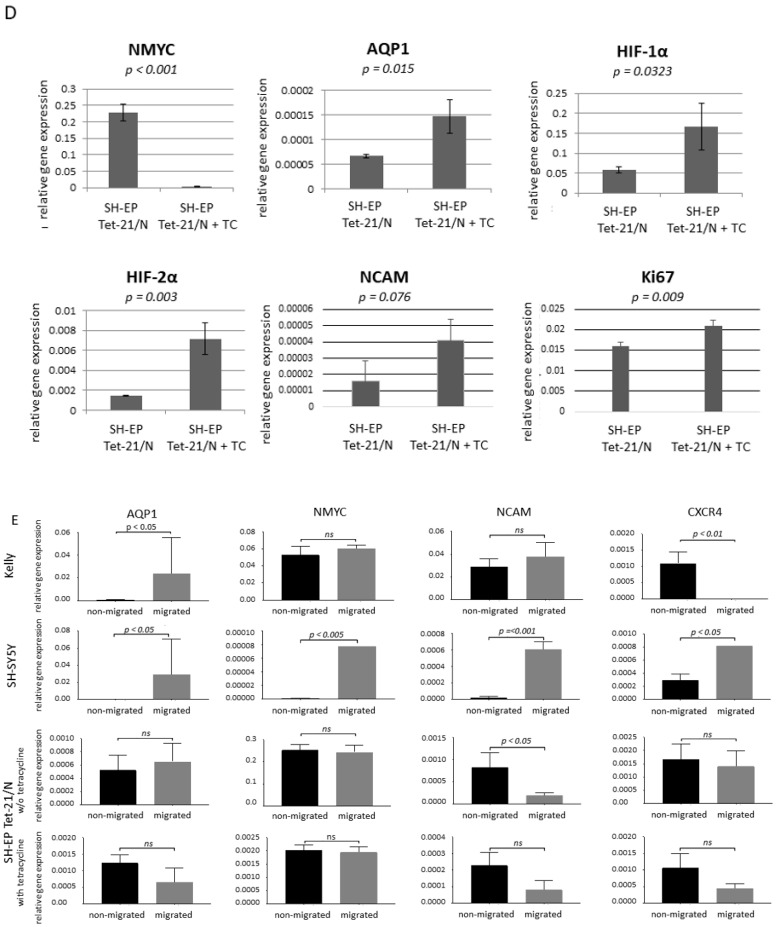
Influence of hypoxia and cell density on AQP1 and on other adverse factors. (**A**) **Up-regulation of AQP1 mRNA under hypoxia.** While AQP1 mRNA is up-regulated under hypoxia (1% O_2_) in Kelly and SH-SY5Y cells, which have a confirmed AQP1 protein expression, NMYC is down-regulated in SH-EP Tet-21/N and Kelly cells, that have an established NMYC amplification. Relative gene expression is depicted in relation to the housekeeping gene 18s (*y*-axis). (**B**) **Up-regulation of AQP1 protein under hypoxia.** Kelly and SH-SY5Y both increasingly express AQP1 under hypoxic versus normoxic conditions as shown in immuno-cytofluorescence staining (AQP1—red, DAPI—nuclei). On the visually distinguishable level this is independent of the density in which they are grown. See Supplementary Material (Figure S1) for a more detailed view. (**C**) **Cell density assay.** When growing SH-EP Tet-21/N, SH-SY5Y and Kelly cells in different cell densities (low, middle and high) intra-cell culture heterogeneity becomes quite obvious. The following cell concentrations were used: low (Kelly and SH-SY5Y-1 × 10^5^ cells/mL; SH-EP Tet21/N cells-1 × 10^4^ cells/mL), middle (Kelly and SH-SY5Y-5 × 10^5^ cells/mL; SH-EP Tet21/N cells-5 × 10^4^ cells/mL), highly (Kelly and SH-SY5Y-1 × 10^6^ cells/mL; SH-EP Tet21/N-1 × 10^5^ cells/mL) dense cell concentrations. Kelly and SH-EP Tet-21/N show distinct cell property differences depending on their density. Cells grown in low densities under normoxia express a hypoxic marker profile with up-regulation of AQP1, HIF-1α and HIF-2α, as well as with up-regulation of the adverse factors NMYC and NCAM. The expression profile of SH-SY5Y cells is less conclusive. CXCR4 expression pattern varies between cell lines. Relative gene expression to 18s is depicted on the *y*-axis. (**D**) **NMYC-related profile in SH-EP Tet-21/N with or without tetracycline.** Relative expression of AQP1, HIF-1α, HIF-2α, NMYC, NCAM and Ki67 mRNA is shown in relation to the housekeeping gene 18s (*y*-axis) with or without treatment with tetracycline, which leads to expression (*w*/*o* tetracycline) or absence (with tetracycline) of NMYC. Similar to our observations under hypoxia, AQP1 regulation is reciprocal to that of NMYC. The cells in which NMYC is absent show an increased hypoxic profile, which is in congruence with up-regulation of AQP1. Interestingly, the absence of NMYC also leads to an increase in the proliferation marker Ki67. (**E**) Expression profile of migrating tumor cells.

Exposing SH-EP Tet-21/N, Kelly and SH-SY5Y cell to hypoxia led to an up-regulation of AQP1 mRNA measured by quantitative PCR, if AQP1 is present in the first place to any relevant degree in those cell line (Figure 2A), as is the case for Kelly and SH-SY5Y cells. Immunofluorescence staining confirmed overexpression of AQP1 in Kelly and SH-SY5Y cells under hypoxic conditions regardless of their seeding concentration (Figure 2B). While we also observed up-regulation of well-known, hypoxia-associated factors and key regulators like HIF-1α and HIF-2α, we found that hypoxia not only altered these known factors, but also led to a decrease in NMYC if cell lines were NMYC positive as seen in the case of SH-EP Tet-21/N and Kelly cells (Figure 2A).

We often observe changes of cell phenotype within one cell culture dish. To study the dependence of cell phenotype on cell density in the culture dish, we quantified this by growing cells at three different concentrations (low, middle and high density) under normoxic conditions. We then examined changes in gene expression of cells that were grown at different densities in the cell culture dish. While finding moderate changes in the expression of AQP1 between different densities, showing a higher AQP1 expression in cells grown at lower density compared with moderately and highly concentrated cells, we found significant differences in the expression of HIF-1α, HIF-2α and NMYC (Figure 2C). The hypoxia key regulators HIF-1α and HIF-2α, as well as NMYC, were up-regulated under seemingly normoxic conditions in Kelly and SH-EP Tet-21/N cells grown at low densities. Data for SH-SY5Y cells mRNA were not conclusive, but AQP1, HIF-1α, HIF-2α and NMYC show an increase when comparing high cell density with low cell density. However, AQP1 protein expression was up-regulated as measured by immunofluorescence staining in hypoxia compared to normoxia in low as well as in higher cell densities in Kelly and SH-SY5Y cells as shown in Figure 2B. The expression of NCAM could not conclusively be connected with any cell density. It appears that cells sown in a medium-density are more likely to express NCAM in Kelly and SH-SY5Y cells. In SH-EP Tet-21/N cells expression levels for NCAM are very low (see scaling of *Y*-axis).

When we compare the cells that actually migrate with the non-migrating cells in the modified transwell assay we observe an increased expression of AQP1 together with an increased expression of NMYC and NCAM at least to some extent in migrating Kelly and SH-SY5Y cells. Relative gene expression to 18s is depicted on the *y*-axis. As AQP1 is almost absent in SH-EP Tet-21/N, we neither see this phenomenon with NYMC nor without NMYC expression. The NCAM expression in SH-EP Tet-21/N again is very low (see values on *y*-axis), but reduced in the migrating tumor cells and independent of NMYC expression.

A factor that is well known to be associated with migration in neuroblastoma is the chemokine receptor CXCR4. In the cell density assay, the expression of CXCR4 is declining towards the low density in Kelly cells which is reciprocal to AQP1 and NMYC expression. In congruence with this, SH-SY5Y cells also showed the lowest CXCR4 expression in low cell density. CXCR4 mRNA levels were extremely low in SH-EP Tet-21/N in the absence of tetracycline (Figure 2C). We previously described CXCR4 protein expression in Kelly and SH-EP Tet-21/N cells [14].

We next used the tetracycline-driven NMYC on/off mechanism of SH-EP Tet-21/N to investigate how the modification of NMYC influences the expression of AQP1 (Figure 2D). AQP1 expression is significantly increased in SH-EP Tet-21/N in which NMYC is switched off. This increase goes along with an increase in the hypoxia-related factors HIF-1α and HIF-2α. Furthermore, an increase in NCAM is observed, again on a very low but significant level. At the same time, we see an increase in proliferation determined by Ki67 expression. Furthermore, we show that in an AQP1 knock-down construct of SH-SY5Y cells NMYC is down-regulated. Expression of NMYC is also compromised under its induction through hypoxia in the absence of AQP1 compared to the wild type cell line.

Our data show that although NMYC is a well-established, clinical marker for tumor cell aggressiveness in neuroblastoma, NMYC is highly dependent on other factors in the heterogeneous presentation of neuroblastoma cells. Our data suggest that AQP1, as a potential enhancer of migration, together with HIF-1α and HIF-2α, as markers of the hypoxic phenotype, and an increase in proliferation are able to modulate NMYC expression.

In a modified transwell assay we investigated which properties are expressed by the cells that actually migrated. We used Kelly cells, which are NMYC and NCAM positive under regular cell culture conditions, and SH-SY5Y cells, which are NMYC and NCAM negative under regular cell culture conditions (Figure 2E). We observe that the cells that actually migrate are prevailingly cells that express elevated levels of AQP1 in Kelly, SH-SY5Y and NMYC expressing SH-EP Tet-21/N cells. NMYC and NCAM are predominantly expressed in migrated Kelly and SH-SY5Y cells, although in the case of SH-SY5Y to a minimal extent regarding low NMYC and NCAM gene expression levels. For SH-EP Tet-21/N cells AQP1 is increased in the migrated cells in the presence but not in the absence of NMYC expression, despite very low gene expression levels. In both SH-EP Tet-21/N cell lines migrated cells express lower levels of NCAM compared to the non-migrating cells.

### 3.3. Differentiation

**Figure 3:**
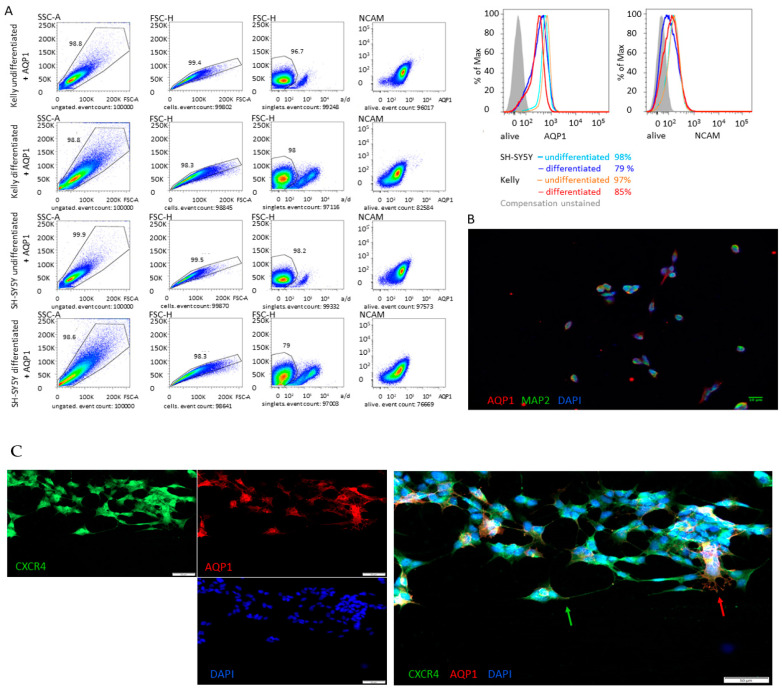
(**A**,**B**) Influence of differentiation on AQP1 expression. (**A**) Differentiated Kelly and SH-SY5Y cells show reduced expression of NCAM as well as of AQP1 protein in FACS anaysis. Cells were gated on single cells, followed by LIVE/DEAD negative living cells, and subsequently analyzed for NCAM and AQP1 expression. Histograms compare fluorescence intensities between groups. (**B**) In immunofluorescence staining differentiated SH-SY5Y cells have lost their «out-spreading» phenotype and have adopted an almost round shape. Compared to undifferentiated cells in Figure 2B and Figure 3C the AQP1 expression of differentiated cells is decreased (AQP1 red color, nuclei are stained in blue and MAP2 in green). (**C**) **AQP1 expression is driving inital migration** Immunofluorescence staining of SH-SY5Y overexpressing cells reveals that during the intial phase of scratch migration assay, AQP1 (red) and not CXCR4 (green) is initializing migration and forming an «out-spreading» phenotype. The green arrow indicates CXCR4 positive filopodia that are directed towards other cells, while the red arrow indicates filopodia of AQP1 expressing cells, that are spreading out towards the cell gap and thus initiate migration of the cell.

We applied a differentiation protocol to SH-SY5Y and Kelly cells simultaneously [3]. After 18 days of this protocol, we saw significant changes in cell properties regarding AQP1 and NCAM protein levels. FACS analysis for SH-SY5Y and Kelly cells showed a decrease in NCAM levels which would be expected in cells with a higher level of differentiation (Figure 3A). It also revealed lower AQP1 expression levels, suggesting that differentiation compromises AQP1 and thus probably AQP1-driven migration. Imaging and co-staining of AQP1 and MAP2 of SH-SY5Y cells after 10 days of the differentiation protocol shows a decrease in filopodia formation and AQP1 expression compared with the usual, extensively spreading-out-pattern of cells (as shown in Figure 2B and Figure 3C) with an increase in MAP2 expression (Figure 3B). Only in a few cells is the typical extension of AQP1 positive filopodia present.

### 3.4. Migration is Independent of CXCR4

We have previously shown that during AQP1-driven migration neuroblasts form filopodia that spread towards the direction in which the cells migrate (Huo to be published). In these filopodia, AQP1 protein is increased to facilitate cell structure re-formation in the migratory progress. In the patient tissue study as well as in the described assays we observed that the expression profile of AQP1 sometimes, but not always coincides with CXCR4, a known migration marker. Meanwhile, in patient 1, tumor piece 2 pronouncedly high AQP1 expression correlates with high CXCR4 and HIF expression, in patient 3, a higher or lower AQP1 expression does not correlate with CXCR4 expression (Figure 1). Something similar is observed in the cell culture in which the CXCR4 expression profile in low, middle and high densities is reciprocal to that of AQP1 in Kelly and SH-SY5Y cells, while correlating in SH-EP Tet-21/N cells despite very low overall expression levels of both genes (Figure 2C).

In order to investigate whether AQP1 expression coincides with CXCR4 expression and if they collude during the migratory process, we used AQP1 overexpressing SH-SY5Y cells in a scratch assay and co-stained for AQP1 and CXCR4 towards the beginning of the migration process. From these pictures, it becomes clear that the mechanical process of forwarding movement through the directional elongation of the filopodia is clearly associated with AQP1 expression, while CXCR4 is more ubiquitously expressed in these cells (Figure 3C). No directional CXCR4-driven elongation of cells can be observed. This clearly suggests an independent mechanism of action for AQP1.

## 4. Discussion

Although we have previously observed that AQP1 expression leads to an increased migratory behavior of neuroblastoma cells through its up-regulation under hypoxic conditions, we observe here that hypoxia leads to a reduction of NMYC in the same cells. A similar effect can be seen when using the tetracycline driven mechanism of SH-EP/Tet cells. When NMYC is not expressed, the expression of AQP1 is increased with increased co-expression of HIF-1α and HIF-2α. We furthermore show that when growing cells in different cell densities, they express these target markers to different degrees. From this, we conclude, that within one cell culture dish there are subsets of cells with higher AQP1 expression. This AQP1 expression correlates with a hypoxic profile of these cells with increased HIF-1α and HIF-2α expression, as well as with NMYC and NCAM expression in two out of three neuroblastoma cell lines. However, when investigating cell properties of actually migrating cells, we find that increased AQP1 expression found in the migrated cells correlates with an increased NMYC and NCAM expression again in two out of three cell lines. AQP1 expression, therefore, correlates with NMYC expression in migrating cells, but correlates with a reduction of NMYC in cells within one stationary cell culture population. Expression of the tumor cell homing marker CXCR4 changes between different tumor areas and between cell lines. While we observe that some migrated tumor cells highly express CXCR4 other cells do not, we determined a dominant role of AQP1 in the migrating cells in the scratch assay.

**Figure 4:**
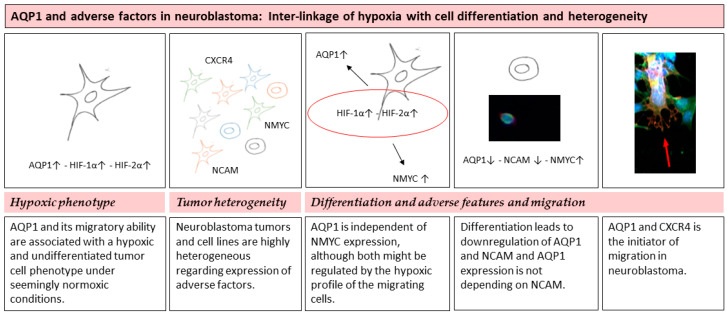
Summarizes the discussion of the three major topics hypoxic phenotype, tumor heterogeneity, differentiation, adverse factors and migration.

We conclude that AQP1 expression is linked to a hypoxic profile as well as it is clearly a driving factor in tumor cell migration. Its relationship with other established adverse factors such as NMYC, NCAM and CXCR4 shows heterogeneity depending on the cell line used as well as the migratory state of the cells. Furthermore, it remains interesting why a subgroup of tumor cells that are grown under seemingly normoxic conditions express the described hypoxic phenotype. Several mechanisms may contribute to these findings. One could be a general intra-tumor or as in our experiments intra-cell culture heterogeneity of a tumor or cell line in culture. Another could be the formation of a cell subpopulation with a hypoxic phenotype with cells that are becoming more eager to metastasize under seemingly normoxic conditions. A third factor contributing to cell migration could be the state of differentiation of a subset of cells. Key findings and discussion topics are schematically summarized in Figure 4.

Figure 4 schematic view of discussion and conclusions.

### 4.1. Tumor Heterogeneity

Neuroblastoma tumors and cells cultures are highly heterogeneous. They originate from different patients’ primary tumor or bone marrow metastases, post-chemotherapy, on resistance, from children of different age groups and different locations with the human body. Furthermore, neuroblastoma is characterized by high intra- and inter-tumor heterogeneity. Neuroblastoma arises from the neural crest, the origin of the sympathetic nervous system. The sympathoadrenal precursor cells are transformed into three major cell lineages, the ganglionic/neuronal, the chromaffin and the small intensely fluorescent lineages [28,29]. The conversion of migrating neural crest cells to sympathetic precursors and finally to differentiated sympathetic neuronal cells has not yet been completely understood. Interruptions of this process however are made accountable for the formation of neuroblastoma. One reason for tumor cell heterogeneity might lie in this process resulting in different neuroblastoma cell subtypes.

It has furthermore been suggested that fluctuations of the cell phenotype result from the kinetics by which cells from different tumors or between different regions within a tumor restore their normoxic phenotype upon reoxygenation after oxygen deprivation due to transient, insufficient oxygen supply [22].

Another hypothesis is that tumors develop in a stepwise manner [30]. The first and very important step in this process is the selection of cells with a survival advantage. This is followed by clonal expansion of this tumor cell population [30]. It is discussed that one of the factors contributing to intra-tumor heterogeneity is an adaptation to hypoxia, which constitutes an advantage for cell survival and which in turn contributes to the development of specific characteristics of the tumor’s hypoxia-associated microenvironment including structural, vascular and immune aspects [30].

### 4.2. Hypoxic Phenotype

Solid tumors are often characterized by rapid tumor growth combined with a poor or non-effective neo-vascularization leading to areas within the tumor that are deprived of oxygen. This leads to the formation of a subset of cells that grow under these hypoxic conditions, which in turn will lead to the selection of cells that are able to survive under these oxygen-deprived conditions. It is hypothesized that this subset of selected tumor cells develops an increased malignant potential. Due to being able to survive under hypoxic conditions, as well as having a poor nutrient supply and low pH, these cells are also less susceptible to radiation and cytotoxic agents [31,32]. These findings can be confirmed by our patient data. All patients show great variation of hypoxia markers between different areas of the tumor. In most patients, their expression correlates with the expression of AQP1. Moreover, we also find a correlation of the hypoxic factors with the adverse prognostic factors NCAM, CXCR4 and NMYC. Most interestingly, we also find graduation of hypoxic marker genes in the cell culture. PO_2_ gradients that occur along the intracellular diffusion distance in cultured cells have long been discussed [33]. However, our findings show that especially cells that are grown in low densities and not the cells that grow in cell dense layers express significantly higher HIF-1α and HIF-2α levels.

Axelson et al. have previously shown that neuroblastoma cells grown at normoxia express HIF-2α protein. The authors conclude that HIF-2α in contrast to HIF-1α is escaping prolylhydroxylase-induced degradation at normoxia. They believe that while HIF-2α mRNA is up-regulated under hypoxic conditions the expression of HIF-1α is almost unaffected by low oxygen [24]. The group has also observed that HIF-2α is already stabilized at 5% oxygen in neuroblastoma cells, while HIF-1α is not [22,34]. They show evidence that HIF-2α could be transcriptionally active at 5% oxygen and that HIF-2α determines a specific phenotype of neuroblastoma cells. However, no exclusive target gene of HIF-2α has been identified yet, while most known genes are regulated by HIF-1α as well as HIF-2α. Studies comparing HIF protein expression with the expression of hypoxia driven genes at the cellular level in tumor sections have shown that HIF-1α/HIF-2α expression is not always congruent with an expression of established hypoxia driven genes such as TH and IGF-2 in neuroblastoma [34]. A similar divergent response has been observed by neuroblastoma cell lines under hypoxia in defined cell culture conditions [35]. The emerging evidence for a diverse role of HIF-1α and HIF-2α could contribute to the unexpected heterogeneity in the hypoxic regulation of specific genes [30]. From these investigations as well as our own data it is apparent that the response of different tumor cells as well as different tumors of the same entity to hypoxia is not always uniform and still poorly understood.

Our data establish a link between the expression of AQP1 and migration, which goes hand in hand with hypoxic expression profile with positivity of HIF-1α and HIF-2α of migrating cells. We furthermore identify a subset of cells in the cell culture under seemingly normoxic conditions that combine these phenotypical properties.

### 4.3. Differentiation and Adverse Features and Migration

Our data demonstrate that the expression of AQP1 is associated with an undifferentiated cell phenotype. With respect to our findings of co-expression of AQP1 and a hypoxic profile, our data are in congruence with findings described in the literature.

Pahlman et al. have shown that neuroblastoma cells, when cultured under hypoxic conditions show many recognized marker genes for neuronal precursor cells, thus become immature and resemble neural crest-derived precursor cells [30]. They hypothesize that the differentiation process is actually driven by hypoxia (24). Jögi et al. have shown that hypoxia causes dedifferentiation both in vitro and in vivo. They propose this to be a mechanism for the selection of highly malignant tumor cells with stem-cell characteristics of neuroblastoma cells. SNS marker genes were down-regulated in hypoxic cells including the lineage-specific transcription factors HASH-1 and dHAND and genes expressed during early neural crest development were up-regulated. The hypoxic cells, therefore, adopted an immature, neural crest-like phenotype [23]. A study by Westerlund et al. has shown that neural differentiation correlates with high levels of HIF-1α but not of HIF-2α, as well as with a better prognosis. They also show that HIF-1α negatively correlates with high-risk features of neuroblastoma, especially NMYC. They suggest HIF-2α to be an unanticipated tumor suppressor in neuroblastoma [21]. Contrarily, another group demonstrated that although HIF-1α is preferentially expressed in NMYC amplified neuroblastoma cells there is no defined regulatory correlation between these two factors [36].

The stress-responsive genetic regulator Sirtuin1, which has been shown to have an influence on the stability of HIF-1α expression has also been associated with the regulation of NMYC expression [36,37,38,39]. In its role as a stabilizer of HIF-1α and HIF-2α expression, it might be hypothezised to be able to contribute to activation of AQP1 in a hypoxia-dependent manner [38,40,41]. This will deserve further studies.

NMYC is known to be of importance during embryonic development [42]. Several birth defects have been linked to a mutation in the NMYC gene such as defects of the central and peripheral nervous system, heart and lung defects, malformations of the genitourinary and intestinal system [43,44,45,46,47]. Interestingly, there seems to be a link between the NMYC pathway and the retinoic acid pathway, as retinoic acid-induced differentiation was shown to be preceded by down-regulation of NMYC [48,49,50]. It has also been suggested that retinoic acid regulates NMYC expression at a transcriptional level [51]. Retinoic acid undoubtedly has a restricting effect on tumor development in vivo, in vitro and in the patient [52,53].

Furthermore, it has been shown that the knockdown of NMYC can lead to neuronal differentiation [54,55,56]. Conversely, this could imply a role of NMYC in maintaining the undifferentiation of neuroblastoma cells [5]. Although data show that NMYC amplification initiates and drives the development of high-risk neuroblastomas, there is evidence that NMYC mRNA may also play an oncogenic role in neuroblastoma that is independent of NMYC protein [57,58,59,60].

The neural cell-adhesive molecule (NCAM/CD56) is highly expressed in neuroblastoma [61,62]. It has been associated with increased malignancy, increased metastatic potential and an overall more aggressive behavior of the tumors leading to a worse prognosis [9,10,11]. However, its expression has proven to be heterogeneous and its function depends on its isoform and its polysialylation status [61,63,64,65]. NCAM is associated with poor differentiation status. Polysialylation of NCAM has been associated with hypoxia [66]. We exposed SH-SY5Y cells to an 18 days differentiation protocol and found NCAM and AQP1 protein to be down-regulated in the differentiation process. This is in congruence with the known data, and confirms that the tumor migration via AQP1 can be down-regulated by differentiation.

The chemokine receptor CXCR4 has been well established as a marker for metastatic homing in various solid tumors including neuroblastoma [12,14]. Expression of CXCR4 in neuroblastoma correlates with a significantly worse outcome compared with a low CXCR4 expression [13]. The process of metastatic homing in this context is driven by the expression of SDF-1α in the homing sites. We previously described a link between PGK1, which is regulated by hypoxia via HIF-1α, to CXCR4 expression [14]. There is also evidence for HIF-dependent activation of CXCR4 under hypoxic conditions in renal cell carcinoma [67]. It still needs to be determined if this hypoxia-associated mode of action is the leading mechanism in neuroblastoma, as CXCR4 is expressed in many different neuroblastoma cell lines under normoxic conditions, Kelly and SH-SY5Y cells amongst them [14].

In this study, we investigated how far the initial migratory process, that we and others have shown to be associated with AQP1, is associated with CXCR4. Our data show that although CXCR4 expression is only partially in congruence with the expression of AQP1, when actually picturing the initial process of migration in the scratch assay, the drivers in the initiation of migration are clearly the AQP1 positive cells.

## 5. Conclusions

Overall, there is a complex inter-linkage of tumor hypoxia with cell differentiation as well as with tumor and cell heterogeneity in neuroblastoma. AQP1 expression and its migratory ability are strongly linked to a hypoxic and undifferentiated profile of the tumor cell even under seemingly normoxic conditions. Neuroblastoma tumors and cell lines are highly heterogeneous regarding the expression of adverse factors. NMYC and NCAM are contributing to migration. However, the expression of AQP1 is independent of NMYC expression, although both might be regulated by the hypoxic profile of the migrating cells. Differentiation leads to down-regulation of AQP1 and NCAM, although again AQP1 expression is not depending on NCAM. AQP1 and not CXCR4 is the initiator of migration in neuroblastoma.

## Data Availability

Data is available on request to the corresponding author.

## Supplementary Materials

The following are available online at https://www.mdpi.com/2227-9067/8/1/48/s1, Figure S1: **MYCN expression SH-SY5Y WT and AQP1 knockdown cells.** The figure shows expression of NMYC mRNA in wildtype and AQP1 knock-down construct of SH-SY5Y cells under normoxia and hypoxia. NMYC is downregulated when AQP1 is knocked down. Furthermore, expression of NMYC is hindered under its induction through hypoxia in the absence of AQP1 compared to the wild type.
