# Supplementary material for: AQP1-Driven Migration Is Independent of Other Known Adverse Factors but Requires a Hypoxic Undifferentiated Cell Profile in Neuroblastoma
Source: Children (Basel). 2021 Jan 15;8(1):48. doi: 10.3390/children8010048 (PMC7829990; doi:10.3390/children8010048)

Supplementary material Figure 2B

Kelly cells
Hypoxia
High density

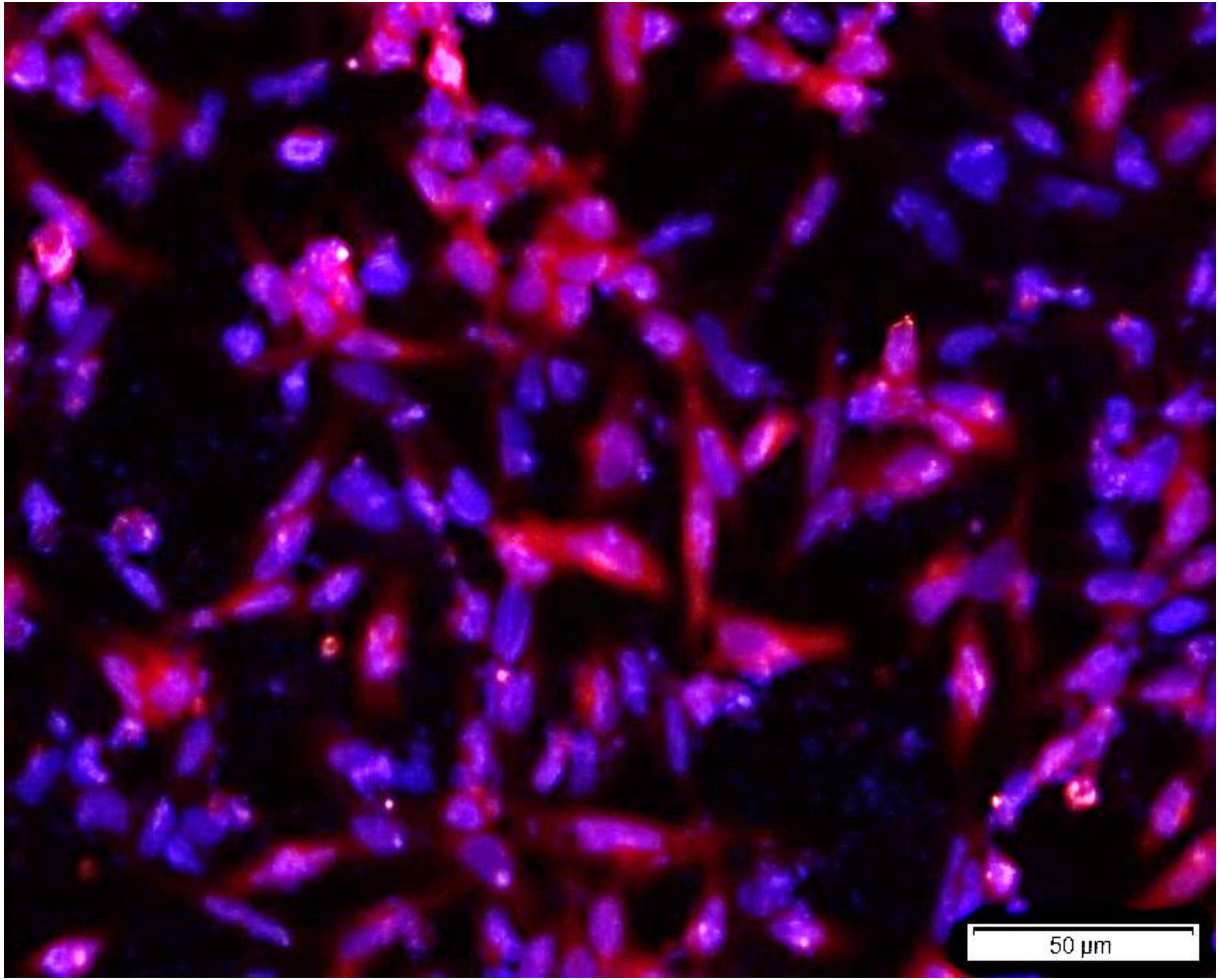

AQP1 DAPI

Kelly cells
Hypoxia
low density

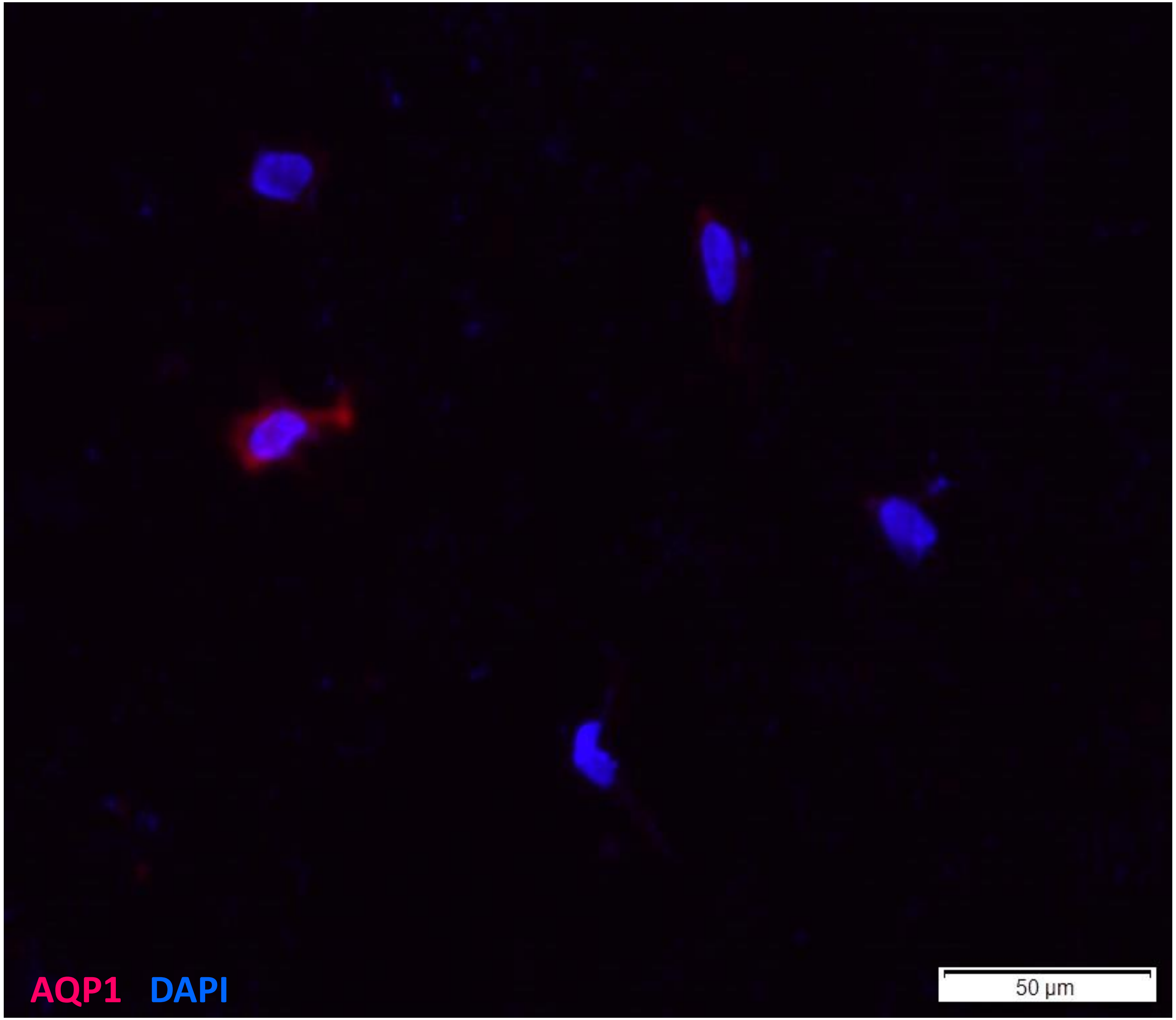

Supplement: Supplementary file 1 [file children-08-00048-s001.pdf]
